# Malignant Paraganglioma With Calvarial Metastases Presenting With Recurrent Catecholamine-Induced Cardiomyopathy

**DOI:** 10.1016/j.aace.2024.09.006

**Published:** 2024-09-30

**Authors:** Beatrice A. Brumley, Run Yu, Shadfar Bahri, Jane Rhyu

**Affiliations:** 1 Division of Endocrinology, Diabetes, and Metabolism, University of California, Los Angeles (UCLA), David Geffen School of Medicine, Los Angeles, California; 2 Division of Nuclear Medicine, Department of Molecular and Medical Pharmacology, University of California, Los Angeles (UCLA), David Geffen School of Medicine, Los Angeles, California

**Keywords:** metastatic pheochromocytoma/paraganglioma (PCC/PGL), calvarial metastases, catecholamine-induced cardiomyopathy, peptide receptor radionuclide therapy (PRRT)

## Abstract

**Background/Objective:**

Cranial metastases rarely occur in malignant paragangliomas (PGLs) or pheochromocytomas, which usually metastasize to the liver, bone, lungs, and lymph nodes. Early detection and intervention with a multidisciplinary approach are crucial given the critical location.

**Case Report:**

Our patient was a 31-year-old man diagnosed with periaortic PGL and succinate dehydrogenase subunit B pathogenic variant at the age of 9 years with cardiac arrest. He developed intra-abdominal and skeletal metastatic disease by the age of 14 years and treated with surgery, chemotherapy, and radiation. After being lost to follow-up, the patient presented emergently with headache, palpitations, hypertensive crisis, type 2 non-ST-elevation myocardial infarction, and catecholamine-induced cardiomyopathy, with plasma free metanephrine level of 61.0 pg/mL (0.0-88.0 pg/mL) and elevated serum free normetanephrine level of 662.9 pg/mL (0.0-210.1 pg/mL). Imaging showed a right frontal calvarial lesion, with 4.9-cm intracranial dural and 4.9-cm extracranial components, and a 1.5-cm occipital bone lesion. Following adrenergic blockade, the patient underwent resection of the frontal lesion with pathology showing metastatic PGL.

**Discussion:**

A multidisciplinary team was consulted. Because of potential neurotoxicity, radiology advised against radiotherapy. Oncology advised monitoring. Seven months postoperatively, gallium-68 dodecane tetraacetic acid–octreotate positron emission tomography/computed tomography showed no recurrence at the surgical site, stable occipital lesion, and additional skeletal metastases. The patient is planned for peptide receptor radionuclide therapy.

**Conclusion:**

Our case highlights the importance of active surveillance in PGL and pheochromocytoma to allow early intervention for metastatic disease and reviews the controversial management of rare calvarial or cerebral metastases, including peptide receptor radionuclide therapy.

**Highlights**

• Paragangliomas (PGLs)/pheochromocytomas (PCCs) need lifelong active surveillance
• Early recognition and intervention of metastatic lesions in PGLs/PCCs are crucial
• Cranial metastases in PGLs/PCCs are rare, and management is controversial
• First-line management for cranial metastases includes surgery if a candidate
• Peptide receptor radionuclide therapy may be a promising therapy

**Clinical Relevance**

Cranial metastases in paragangliomas/pheochromocytomas are exceedingly rare. Lifelong, active surveillance is key in allowing early recognition and timely intervention for metastatic disease. Limited case reports suggest surgery as first-line treatment in cranial metastases and show potential favorable outcomes for peptide receptor radionuclide therapy, whereas other systemic and radiation therapy are more controversial.

## Introduction

Paragangliomas (PGLs) and pheochromocytomas (PCCs) are neuroendocrine tumors (NETs) that derive from chromaffin tissue in sympathetic or parasympathetic paraganglia and the adrenal medulla, respectively.^1^^,^^2^ Their malignant potential is difficult to predict via biochemical, histopathologic, or genetic parameters, with malignancy diagnosed retrospectively after metastasizing.^1^^,^^2^ Approximately 10% of PCCs and 35% of PGLs are malignant,^3^^,^^4^ and metastases can occur >50 years after diagnosis, necessitating long-term follow-up.^4^ Behavior of malignant PGL/PCC is widely variable, from mortality within a year to stable metastatic disease for >40 years.^4^

Cranial metastases are rarely seen in PGLs/PCCs,^5^, ^6^, ^7^, ^8^ which typically metastasize to the liver, bone, lungs, and lymph nodes.^1^^,^^7^ Calvarial or cerebral metastases in PCCs have occurred in approximately 20 cases, and outcomes vary from rapid mortality to survival >5 years after resection.^5^, ^6^, ^7^, ^8^ Management and prognosis remain controversial with high risk, due to the critical location.^5^, ^6^, ^7^, ^8^

## Case Report

Our patient was a 31-year-old man diagnosed with periaortic PGL at the age of 9 years after a cardiac arrest while watching television, followed by resection. Genetic testing revealed a succinate dehydrogenase subunit B (*SDHB*) pathogenic variant. Although his parents did not have NETs, his brother and maternal grandmother were diagnosed with recurrent neck PGLs and a carotid body tumor, respectively.

The patient developed metastatic PGL by the age of 14 years, when he presented with lightheadedness and diagnosed with intra-abdominal metastases to the spleen and left periadrenal gland and skeletal presacral and left femoral metastases. He underwent resection of the intra-abdominal lesions and chemotherapy with temozolomide and thalidomide. Over the ages of 17 to 19 years, he developed metastases to the scapula, inferior retroperitoneum, aortic bifurcation, and T6 to T9 vertebrae and underwent retreatment with temozolomide, thalidomide, and bevacizumab until the age of 22 years, along with radiation for vertebral metastases. His last imaging noted fluorine-18 (^18^F) fluorodeoxyglucose (FDG) avidity within the metastatic skeletal, retroperitoneal, and aortocaval sites, without uptake on metaiodobenzylguanidine scan. The patient reported lack of follow-up for >5 years with the last urine catecholamine level normal per recall.

The patient presented to an outside hospital after waking up overnight with an acute headache, with palpitations and headaches developing over 6 months and a skull mass subjectively noted for a few days. The patient was hypertensive and provided intravenous labetalol, precipitating hypertensive crisis and seizures. He was started on levetiracetam and intubated. He subsequently became hypotensive, started on pressors, and diagnosed with a type 2 non-ST-elevation myocardial infarction from demand ischemia and nonischemic cardiomyopathy (NICMY) with a left ventricular ejection fraction of 25% to 30%. Laboratory results showed a plasma free metanephrine level of 61.0 pg/mL (0.0-88.0 pg/mL) and serum free normetanephrine level of 662.9 pg/mL (0.0-210.1 pg/mL). The patient was started on alpha-adrenoreceptor blockade, followed by extubation, and transitioned to our center for higher level of care.

**Fig. 1:**
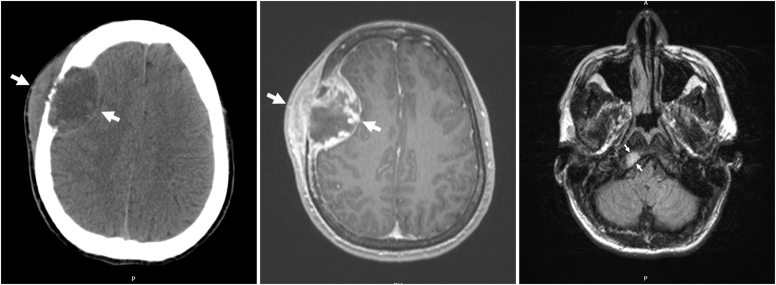
Imaging findings on presentation. (Left) Computed tomography of the brain without contrast showed a destructive lesion (wide arrows) centered in the right frontal bone with associated intracranial and extracranial extensions. The intracranial dural-based component measured 4.9 × 3.2 cm, and the extracranial component measured 4.9 × 1.3 cm. There was a leftward midline shift of 6 mm due to mass effect. (Middle) Magnetic resonance imaging with gadolinium confirmed the right frontal lesion (wide arrows), which exhibited intense enhancement. (Right) Magnetic resonance imaging T2 fluid-attenuated inversion recovery protocol revealed a second lesion in the right occipital bone, which measured 1.5 cm (thin arrows).

At our center, the patient’s vital signs were normal, and examination was notable for a 5-cm right frontal mass. Computed tomography (CT) of the brain showed a destructive lesion in the right frontal bone, with intracranial dural-based component measuring 4.9 cm and extracranial extension measuring 4.9 cm, associated edema, and 6-mm leftward midline shift (Fig. 1). Brain magnetic resonance imaging (MRI) showed intense enhancement of the right frontal lesion and revealed a 1.5-cm right occipital calvarial lesion (Fig. 1).

Ten days after presentation, the patient underwent craniotomy and resection of the right frontal calvarial mass. Pathology was consistent with metastatic PGL (Ki-67 staining of 5%). Prior to surgery, doxazosin was uptitrated to 4 mg twice daily, and metoprolol tartrate was subsequently added and uptitrated to 75 mg twice daily. Preoperative cardiac workup showed normalized left ventricular ejection fraction and a normal CT coronary angiogram. Despite preoperative high salt diet and intravenous fluid resuscitation, the patient experienced intraoperative hypotension and briefly required pressors postoperatively. Because of suspicion for persistent catecholamine secretion given the occipital calvarial tumor and known metastases, he was discharged by postoperative day 4 on doxazosin 0.5 mg twice daily and metoprolol tartrate 12.5 mg twice daily with normal vital signs.

**Fig. 2:**
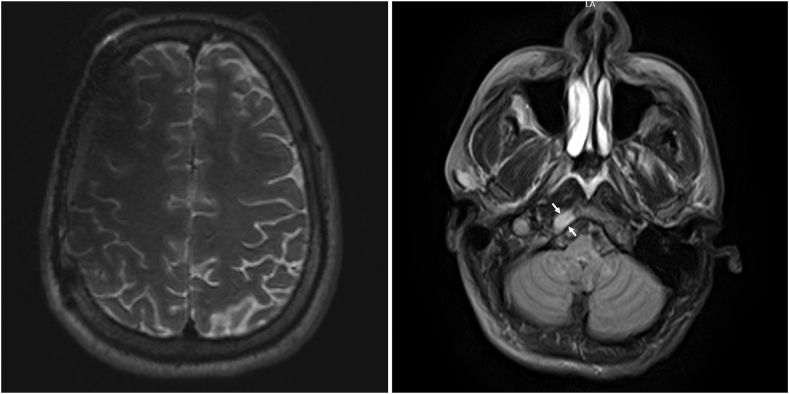
Magnetic resonance imaging findings 3 months after the resection of the right frontal lesion. (Left) The right frontal lesion was no longer seen. (Right) The right occipital bone lesion persisted but remained unchanged in size (thin arrows).

**Fig. 3:**
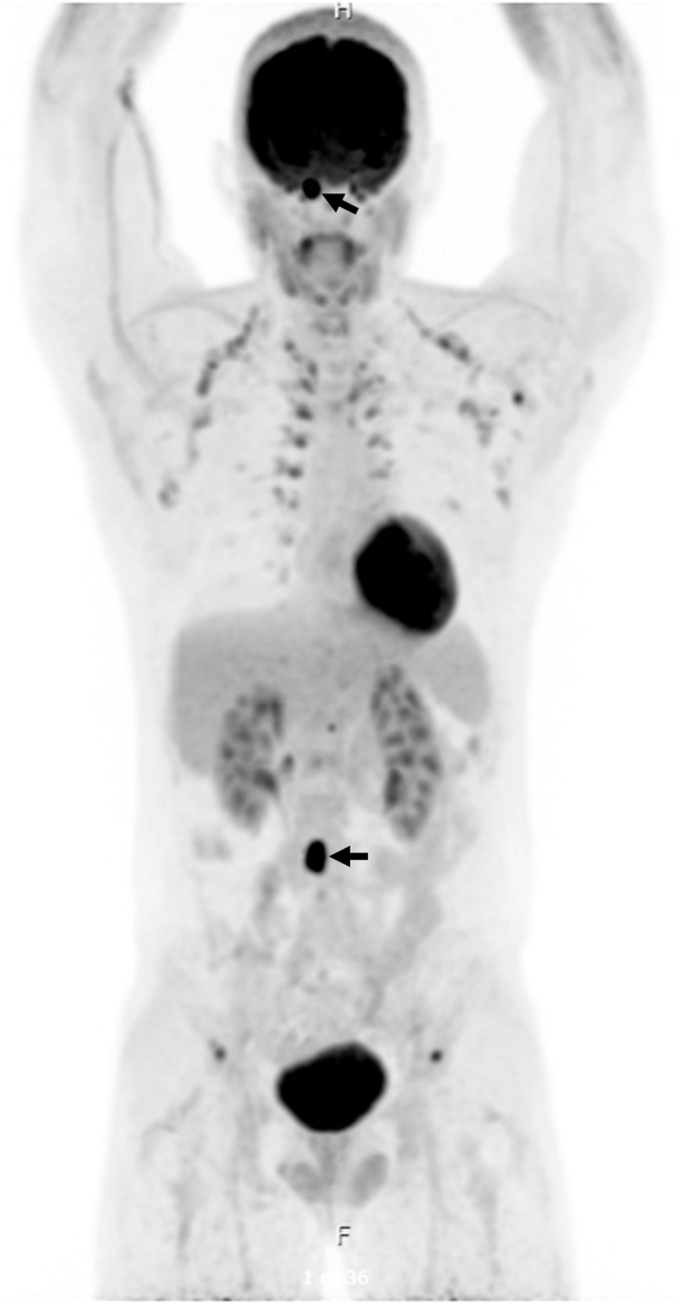
Fluorine-18 fluorodeoxyglucose positron emission tomography 4 months after resection showed extensive bony metastases, including the lesion in the right occipital bone and a prevertebral lesion (arrows).

**Fig. 4:**
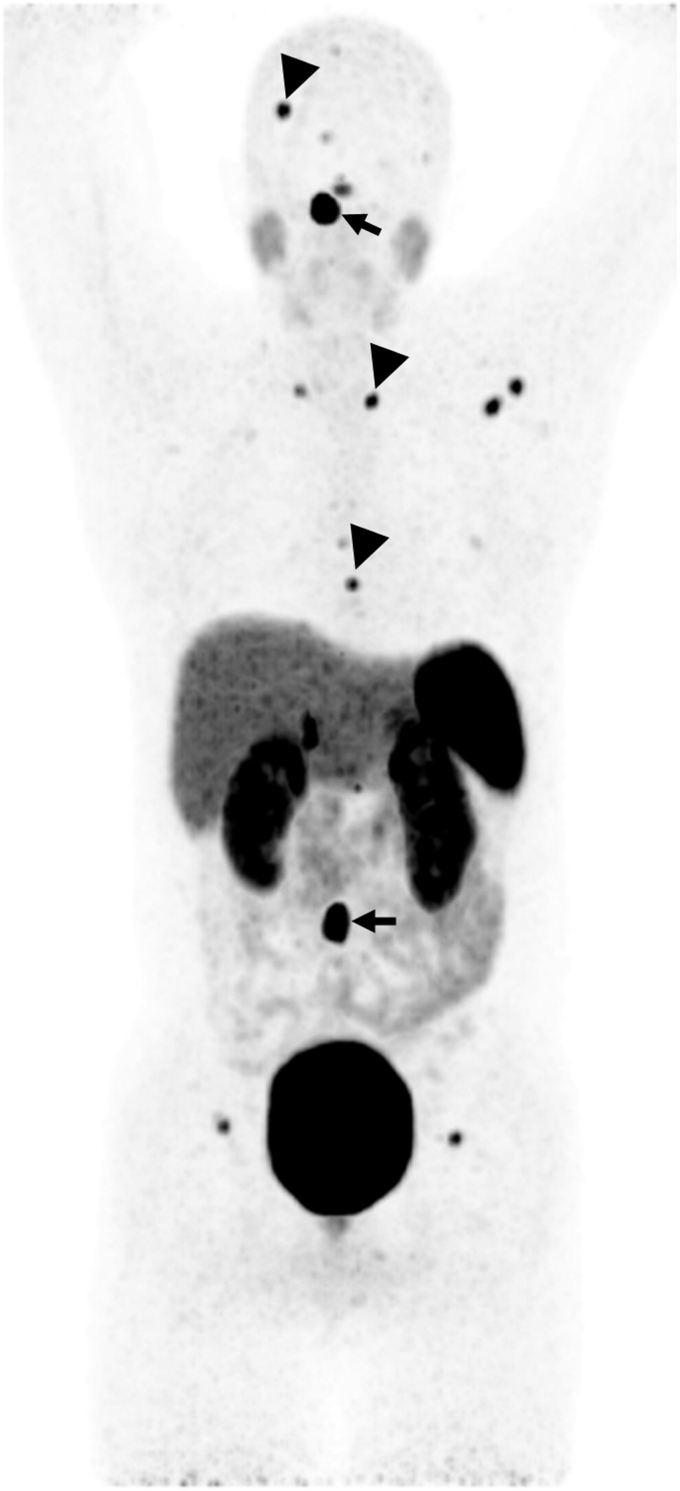
Dodecane tetraacetic acid–octreotate (DOTATATE) positron emission tomography (PET) 7 months after resection confirmed extensive bony metastases, including the lesion in the right occipital bone and a prevertebral lesion (arrows). In addition, the DOTATATE-PET showed additional lesions (arrowheads) that were not obvious on the fluorodeoxyglucose PET, which was likely due to the superior sensitivity of DOTATATE-PET rather than true new lesions.

Postoperative imaging at 3 to 4 months showed no evidence of residual tumor at the surgical site on brain MRI (Fig. 2), and ^18^F-FDG positron emission tomography (PET) showed widespread bony metastases, including the occipital lesion (Fig. 3). Postoperative laboratory results at 5 months showed a plasma free metanephrine level of 39 pg/mL (normal, <57 pg/mL) and plasma free normetanephrine level of 253 pg/mL (normal, <148 pg/mL). Gallium-68 dodecane tetraacetic acid–octreotate (^68^Ga-DOTATATE) PET/CT at 7 months confirmed uptake at the known metastatic sites (Fig. 4).

We are now pursuing treatment with peptide receptor radionuclide therapy (PRRT) with lutetium Lu 177 dotatate (LUTATHERA). Because of potential neurotoxicity, radiation oncology recommended holding off radiation, unless there are concerns for residual disease or regrowth. Hematology/oncology advised monitoring with ^18^F-FDG-PET every 6 months and alternative axitinib or lenvatinib therapy.

## Discussion

The key toward PCC/PGL management relies on surveillance and timely intervention for critical presentations—especially pertinent in patients presenting in childhood.^7^ Ten to twenty percent of PCCs/PGLs occur in the pediatric population, with germline pathogenic variants in up to 80% of pediatric cases.^9^

The *SDHB* gene encodes succinate dehydrogenase subunit B, an enzyme involved in the mitochondrial tricarboxylic acid cycle.^10^ Pathogenic variants of *SDHB*, as in our patient, lead to pheochromocytoma/paraganglioma syndrome type 4 (PGL4). PGL4 has an autosomal dominant inheritance pattern with 20% to 30% disease penetrance by the age of 65 years. First-degree relatives should be offered genetic testing and undergo biochemical or imaging surveillance if positive.^10^^,^^11^ Associated PCCs/PGLs have a 35% to 40% lifetime metastasis risk,^10^ and the median survival after diagnosis is 28 years.^9^ Because of association of *SDHB* variants with metastases, all patients with metastatic PCC/PGL should be tested for *SDHB* variants.^11^ Patients with PGL4 additionally have an increased risk of renal cell carcinomas, pituitary adenomas, and gastrointestinal stromal tumors.^10^

Surveillance in PCC/PGL is lifelong and crucial with germline pathogenic variants.^10^^,^^11^ Patients should receive whole-body imaging and biochemical surveillance approximately every 1 to 2 years, with closer monitoring after diagnosis or surgery and less frequent surveillance after stability.^10^ In *SDHB* pathogenic variants, the highest metastatic risk occurs within 2 years after diagnosis and 12 to 18 years after diagnosis (reflecting our patient’s progression), with heightened surveillance considered then.^9^

Management of metastatic PCC/PGL centers on addressing functional and structural components.^3^ Biochemical excess is treated with long-term adrenergic blockade to prevent complications of catecholamine excess.^3^ Somatostatin analogs are controversial in effectiveness.^3^ Treatments for metastatic sites range widely, with surgery considered first-line;^3^^,^^12^ other options include radiation, ablation, embolization, radionuclide therapy, chemotherapy, immunotherapy, and molecular targeted therapy.^3^^,^^12^

For our patient, surveillance may have prevented a critical presentation. Cardiac complications of PCC/PGL are feared (including arrhythmia, myocardial infarction, ischemic cardiomyopathy, NICMY, and aortic dissection), presenting emergently as hypertensive crisis, cardiogenic shock, cardiac arrest, or death.^13^, ^14^, ^15^, ^16^ Treatment involves close management with cardiology. Careful alpha-adrenoreceptor blockade is key in addressing catecholamine excess, reversing our patient’s NICMY.^13^, ^14^, ^15^, ^16^ Beta-adrenoreceptor blockade should not be employed without adequate alpha-adrenoreceptor blockade. Doing so may precipitate a hypertensive crisis,^17^ as our patient experienced after labetalol use, a nonselective adrenoreceptor antagonist with more potency at the beta-receptors than at the alpha-receptors.

Once medically stabilized, surgery would be the treatment of choice, which is potentially curative for cardiac complications of PCC/PGL and can resolve even subclinical endocardial dysfunction.^13^, ^14^, ^15^, ^16^ If cardiomyopathy persists, guideline-directed medical therapy for heart failure can be employed.

After neurosurgery deemed the dominant frontal lesion amenable for surgery, we agreed with surgical management. A review of PCCs with cerebral or calvarial metastases showed favorable surgical outcomes in 9 of 12 patients, improving functional prognosis, quality of life, and possibly survival.^5^^,^^8^ Surgical debulking also relieves mass effect (notably if edema or focal neurologic deficits are present), reduces biochemical burden, and improves efficacy of subsequent therapies.^3^^,^^12^

Along with experienced neurosurgery and anesthesia care, preoperative care is paramount to stabilize hemodynamics and maximize surgical and neurologic outcomes. Adrenergic blockade is crucial and reasonable even in biochemically silent tumors as performed in a PCC case with cerebral metastasis;^8^ intraoperative catecholamine excess has occurred with manipulation and anesthesia in silent tumors.^18^ Preoperative endovascular ablation can be considered given tumor hypervascularity.^5^^,^^8^ Fluid resuscitation and high salt load, as tolerated, are important due to the intravascular depleted state in PCC/PGL.^5^^,^^8^ Our patient’s intraoperative hypotension would have been more profound without intensive preoperative alpha-adrenoreceptor blockade and fluid replenishment. Our patient underwent resection of the frontal lesion with a favorable outcome consistent with the literature.

Given the high risk of calvarial metastases and the patient’s history of mass effect and seizures, we are planning for PRRT as adjunct therapy for the resected site and treatment for the occipital lesion and other metastatic sites. PRRT, which delivers selective radiation via radiolabeled somatostatin analogs, has shown promising results. Despite concerns of PRRT’s feasibility to cross the blood-brain barrier,^19^ case reports of NETs with cerebral metastases showed improved progression-free survival (>3 years in 1 study) but mild impact on tumor volume.^19^^,^^20^ PRRT was provided with dexamethasone because of potential intracranial edema.^19^^,^^20^ Other treatment options are controversial. Outcomes for radiation varied from near-complete response to complications with worsening cerebral edema and radiation necrosis.^5^^,^^6^^,^^8^ Although chemotherapy is considered treatment of choice for nonsurgical candidates, not enough studies have evaluated efficacy and blood-brain barrier penetration for brain metastases.^5^ Our radiology and hematology/oncology teams independently recommended holding off radiation or systemic therapy, which remain ancillary options. Because of the complexity in metastatic PCC/PGL, a personalized, multidisciplinary approach should be employed.

## Disclosure

The authors have no conflicts of interest to disclose.
